# Idiopathic Benign Impulsive Bilomas

**DOI:** 10.7759/cureus.18099

**Published:** 2021-09-19

**Authors:** Venkata Vinod Kumar Matli, Zachary Shepherd, Amina Dhahri, Kapil Kohli, Raja Sekhar Vadlamudi

**Affiliations:** 1 Internal Medicine, State University of New York Upstate Medical University, Syracuse, USA; 2 Internal Medicine, University of Maryland Capital Region Medical Center, Largo, USA; 3 Internal Medicine, University of California San Francisco Medical Center, San Francisco, USA; 4 Gastroenterology and Hepatology, Wake Endoscopy Center, Raleigh, USA

**Keywords:** perihepatic ascites, biloma, multiple bilomas, biliary ascites, laparoscopic cholecystectomy complication, cystic duct injury, spontaneous bile leak, impulsive biloma

## Abstract

“Biloma” is a collection of bile outside of the biliary tree that could occur postoperatively in patients who undergo laparoscopic cholecystectomy or in patients with blunt trauma to the liver. Spontaneous or impulsive bilomas with no identifiable cause occur rarely. We report a case of a 60-year-old woman with no history of hepatobiliary surgery or trauma, who was admitted for right upper quadrant pain. An abdominal examination revealed tenderness in the right upper quadrant (RUQ). Her alkaline phosphatase level was 2,343 IU/L. Computed tomography of the abdomen and pelvis with contrast showed perihepatic, periduodenal, and right paracolic gutter ascites. The image-guided aspiration of the peritoneal cavity yielded greenish fluid that was determined to be bile. The cytological studies were negative for malignancy and microorganisms. The ultrasound images of the RUQ were negative for cholecystitis and gallstones, and the results of the hepatobiliary nuclear scan study (HIDA) were unremarkable. Magnetic resonance cholangiopancreatography (MRCP) revealed an intact intrahepatic and extrahepatic biliary tree and confirmed the presence of multiple lakes of bile ascites.

During the entire hospital stay, the patient remained stable without any unifying diagnosis and she was discharged with a pigtail catheter. A follow-up abdominal CT scan revealed a complete resolution of the bilomas. We consider this as a spontaneous extra- and intrahepatic biloma of unknown etiology and should be in our differentials when a patient presents with right upper quadrant abdominal pain.

## Introduction

Bile produced in the liver is secreted into the biliary tree, concentrated and stored in the gallbladder, and released into the intestine as needed. A biloma is a collection of bile outside the biliary tree, which may be further classified as extra- or intrahepatic. The term “biloma” was introduced in 1974 by Gould and Patel [1]. Bilomas that occur after laparoscopic cholecystectomy [2] due to bile leakage from the stump or following blunt trauma to the liver are called “intentional.” Meanwhile, “spontaneous” [3] or “unintentional” bilomas, which do not have an identifiable cause, occur rarely. Bilomas are usually benign; however, if not diagnosed and managed early, they may be frustrating for physicians, particularly if a cause cannot be identified. Spontaneous bilomas are rare in our practice, and, as per our knowledge, this was a rare case of a patient with a biloma that presented as right upper quadrant pain with no identifiable cause. The clinical characteristics and rarity make this case important and interesting.

## Case presentation

**Figure 1:**
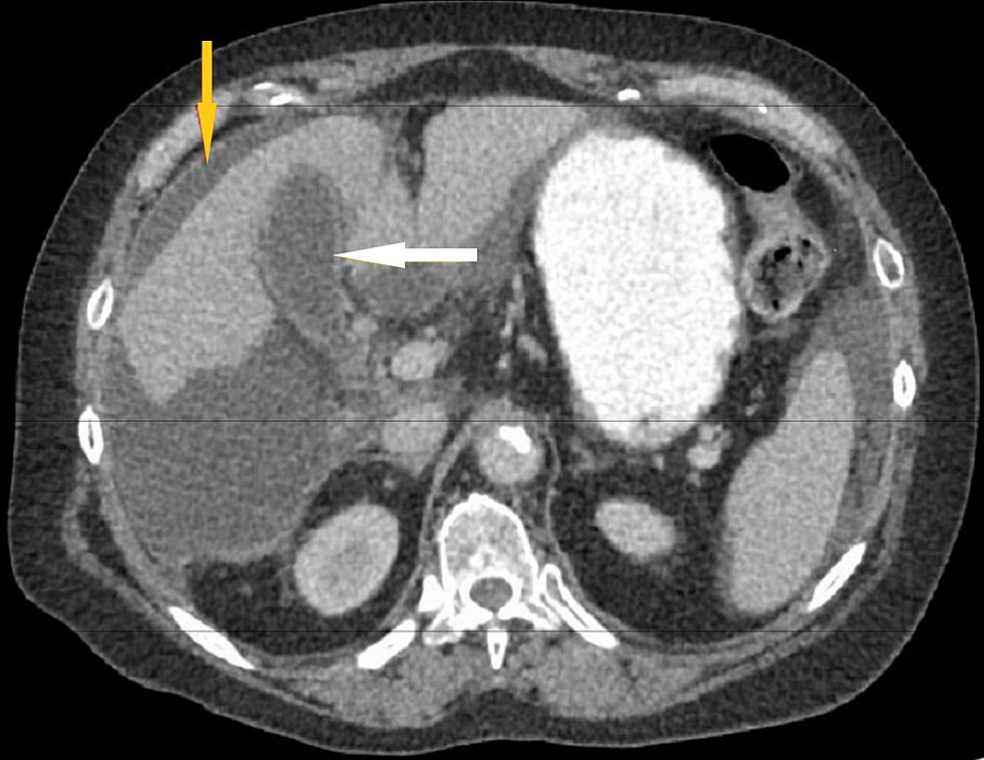
Computed tomography scan of the abdomen and pelvis with contrast. The white arrow shows an intact gallbladder without gallstones or evidence of inflammation. The yellow arrow shows fluid from the extrahepatic biloma, tracking along the anterior and lateral hepatic margins.

**Figure 2:**
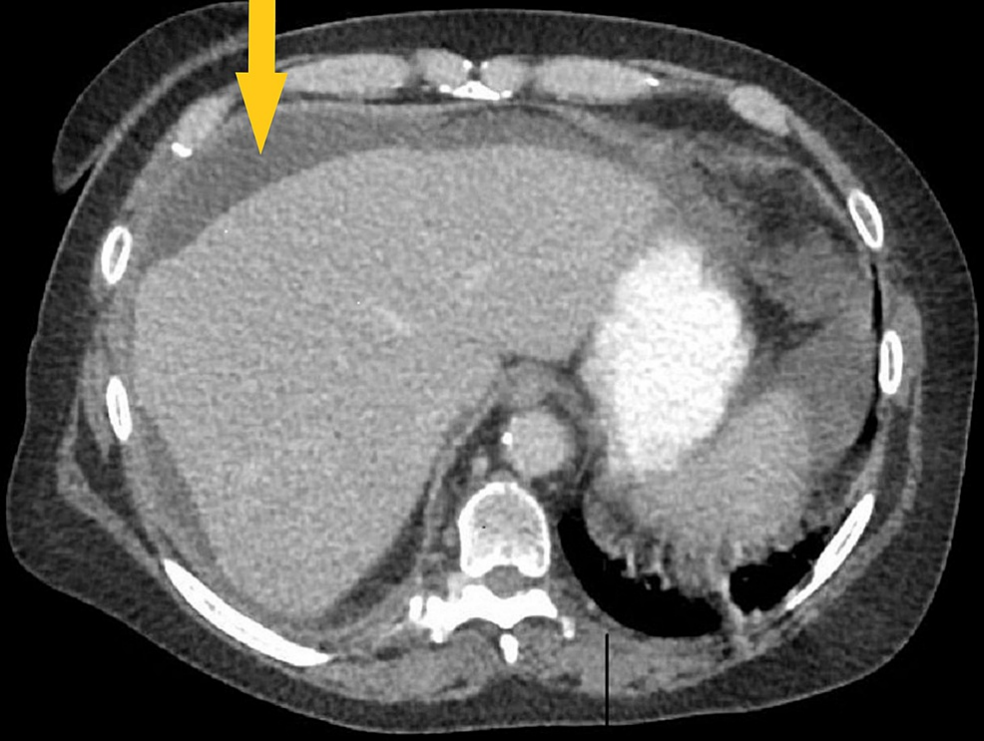
Computed tomography scan of the abdomen and pelvis with a loculated fluid collection measuring 17.8 × 13.4 × 16.7 cm, which is perihepatic bile ascites that has formed an extrahepatic biloma distorting the hepatic parenchyma.

**Figure 3:**
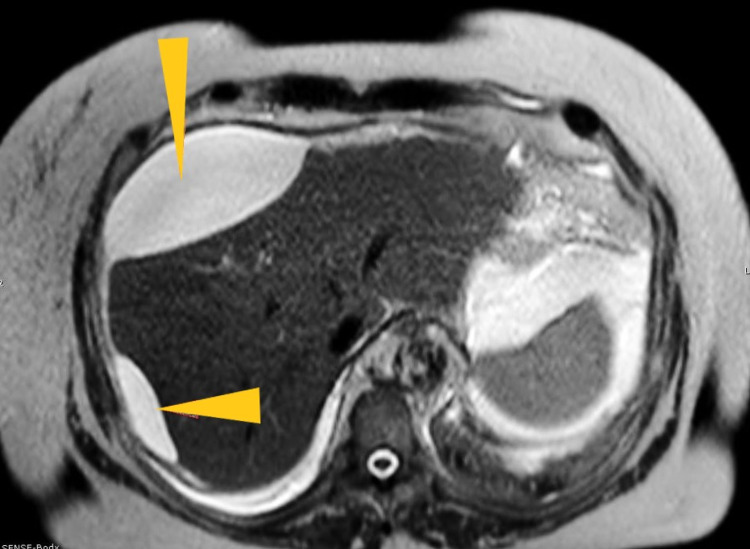
Magnetic resonance image of the biliary tree showing perihepatic bile ascites causing mass effect on the liver.

**Figure 4:**
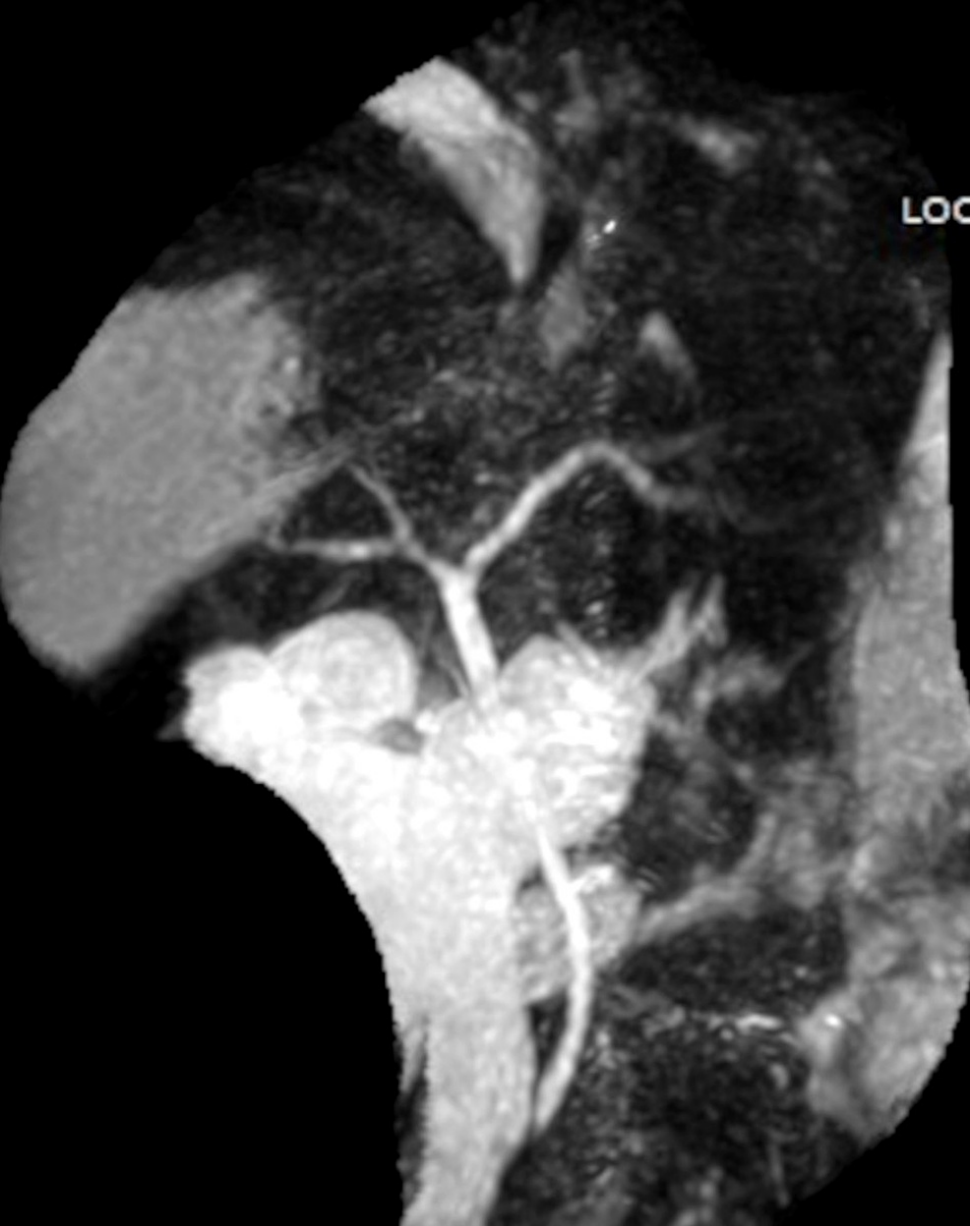
Magnetic resonance image of the biliary tree showing an intact intra- and extrahepatic biliary tree without stones or strictures.

A 60-year-old woman with a medical history of laryngeal squamous cell carcinoma s/p total laryngectomy presented to the emergency department with worsening right upper quadrant (RUQ) pain for four days. There were no other associated symptoms. The pain was sharp, constant, and intermittently shooting up the right side of the body. Nothing made the symptoms worse, and they were not related to food. The patient smoked half a pack of cigarettes daily for 40 years and consumed one or two alcoholic drinks weekly. The family history was notable for lung and pancreatic cancers in her father and mother, respectively. The patient’s vital signs were stable. She was anicteric, with mild distress from the RUQ pain. The cardiopulmonary examination was benign; the abdomen was soft, with mild RUQ tenderness with guarding but no rigidity or rebounding. Normal bowel sounds were heard in all four quadrants. Initial laboratory results revealed an elevated alkaline phosphatase level of 1,961 IU/L and gamma-glutamyltransferase levels were 260 U/L. The levels of bilirubin, transaminases, amylases, and lipases were all within the normal range. Computed tomography (CT) of the abdomen and pelvis with contrast (Figure 1) revealed perihepatic and significant periduodenal ascites in the second part of the duodenum extending along the right paracolic gutter and into the pelvis (Figure 2). The liver, gallbladder, kidneys, spleen, and pancreas were normal, with no evidence of bowel perforation. Following consultation, the surgery team ruled out surgical intervention; however, the gastroenterology team suspected malignancy. Image-guided aspiration of the peritoneal cavity yielded 800 ml of greenish fluid that was determined to be bile. The cytology results and cultures of the bile were negative for malignancy and microorganisms, respectively. The ultrasound images of the RUQ were negative for cholecystitis and gallstones, and the results of the hepatobiliary nuclear scan study were unremarkable. Magnetic resonance cholangiopancreatography (Figures 3, 4) revealed perihepatic bile ascites causing mass effect on the liver and an intact intrahepatic and extrahepatic biliary tree and confirmed the presence of perihepatic, perisplenic, and periduodenal biliary ascites extending downward along the paracolic gutters and insinuating between small bowel loops. The alkaline phosphatase level reached a maximum of 2,343 IU/L on day two and decreased to 533 IU/L at the time of discharge on day 20. The autoimmune workup including antinuclear antibody (ANA), antimitochondrial antibody (AMA), antimitochondrial antibody-M2 (AMA-M2), anti-smooth muscle antibody (ASMA), antineutrophil cytoplasmic antibody (ANCA), tumor markers, QuantiFERON, hepatitis panel, and blood cultures were all negative.

**Figure 5:**
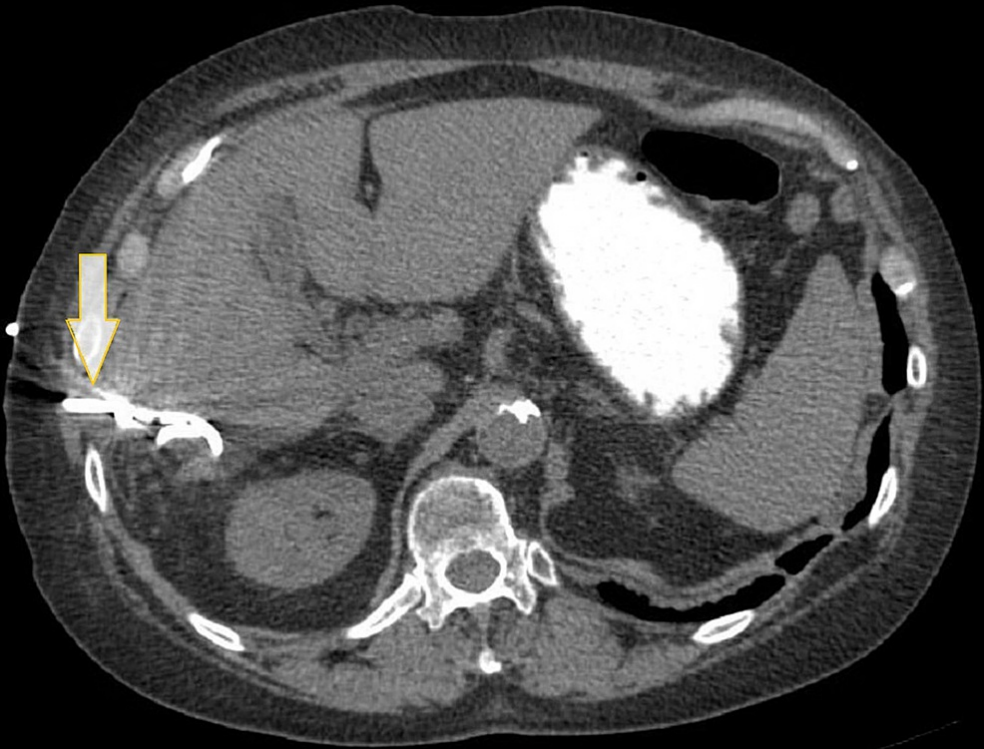
Follow-up computed tomography scan of the abdomen and pelvis without contrast shows almost complete resolution of the biloma. The arrow shows a subhepatic pigtail drain.

Esophagogastroduodenoscopy revealed lymphangiectasia in the second portion of the duodenum and a non-bleeding gastric ulcer in the prepyloric stomach, which underwent biopsy with negative results. An attempted endoscopic ultrasound with retrograde cholangiopancreatography (ERCP) to image the pancreas was unsuccessful because of stenosis of the upper esophagus, which was dilated using a 13.5 mm dilator. During the entire hospital stay, the patient remained stable without any unifying diagnosis, and the patient was eventually discharged on day 20 with a pigtail catheter in place. On follow-up, the patient was doing well clinically. A follow-up abdominal CT scan (Figure 5) revealed complete resolution of the biloma. Elective cholecystectomy was planned because the surgery team felt that the micro-perforations of the gallbladder that spontaneously closed may have caused the bile leak. The surgery was scheduled despite two unsuccessful repeat ERCP procedures.

## Discussion

There are three important points one should know when dealing with bilomas. First, intentional bilomas are common after laparoscopic cholecystectomy or blunt trauma to the liver. In this patient, the cause of the leak still could not be determined; however, micro-perforations of the gallbladder with spontaneous or intermittent sealing remain a possible cause. It was frustrating not to find the definitive cause of the biloma despite an extensive workup on the patient, and it was definitely a challenge for the physician. Only a few cases of spontaneous bilomas of unknown etiology have been reported in the literature. There have been case reports in the literature where the etiology of the bilomas was not identified [4]. There has been a reported case in the literature in which a patient developed spontaneous biloma in the setting of choledocholithiasis. Patients showed resolution of bilomas with CT-guided drain of biloma followed by ERCP with sphincterotomy and extraction of biliary stones [5].

Second, bile peritonitis [6] secondary to a sudden concentrated bile flow into the peritoneal cavity could potentially cause electrolyte abnormalities and hypovolemic shock, which has high morbidity and mortality if not diagnosed and managed promptly and appropriately. Fortunately, our patient showed good clinical improvement and the bilomas were resolved with a pigtail catheter. 

Third, bilomas most often have a benign course, but it is important to identify the cause of the bile leak. As the final diagnosis could not be made in this patient, every step was taken to rule out potential malignancies and possible emergent and dangerous complications. Transcutaneous drainage [7] is a good option to manage large bilomas. Until today, management of bilomas, irrespective of etiology, has been radiological, which involves percutaneous drainage of biliary leaks in the abdomen. Whereas small, benign bilomas can be observed clinically. ERCP is indicated in cases where radiological treatment fails and bile leak persists (recurrent bilomas); this is to divert the flow of bile from the leak site by endoscopic manipulation of the sphincter, which includes sphincterotomy with stent placement, which is required to facilitate bile drainage [8,9].

## Conclusions

Impulsive or spontaneous bilomas are rare, and the cause most often cannot be identified. Periduodenal, perisplenic, paracolic bilomas, and even subscapular bilomas can occur, but our patient may be one of the rare cases of spontaneous multiple bilomas of this type. Bilomas should be one of the differential diagnoses in patients presenting with abdominal pain even without a history of cholecystectomy or blunt trauma to the liver. We term this as “idiopathic impulsive bilomas” as no causes have been identified.
